# Digital empowerment on hold: DiGA adoption gaps−a German national cross-sectional patient survey study

**DOI:** 10.1007/s00296-025-05922-3

**Published:** 2025-07-04

**Authors:** Phillip Kremer, Daniel Fink, Harriet Morf, Hannah Labinsky, Karolina Gente, Peer Aries, Martin Krusche, Felix Muehlensiepen, Sebastian Kuhn, Axel Hueber, Johannes Knitza

**Affiliations:** 1 Division of Rheumatology and Systemic Inflammtory Diseases, III. Department of Medicine, University Medical Center Hamburg-Eppendorf, Hamburg, Germany; 2 Rheumazentrum Mittelhessen, Bad Endbach, Germany; 3 Department of Medicine 3 - Rheumatology & Immunology, Friedrich- Alexander-Universität (FAU) Erlangen-Nürnberg and Uniklinikum Erlangen, Erlangen, Germany; 4 Department of Internal Medicine 2, Rheumatology/Clinical Immunology, University Hospital Würzburg, Würzburg, Germany; 5 Internal Medicine V, Hematology, Oncology and Rheumatology, Heidelberg University Hospital, Heidelberg, Germany; 6 Department of Rheumatology, Immunologikum, Hamburg, Germany; 7 Center for Health Services Research, Faculty of Health Sciences, Brandenburg Medical School Theodor Fontane, Rüdersdorf, Germany; 8 School of Medicine, Institute for Digital Medicine, Philipps-Universität Marburg, Marburg, Germany; 9 Department Internal Medicine 5, Division of Rheumatology, Klinikum Nuremberg, Paracelsus Medical University, Nuremberg, Germany

**Keywords:** Surveys and questionnaires, Digital health applications, Digital therapeutics, Patient self-management

## Abstract

Digital health applications (DiGAs), prescribable and reimbursed in Germany since 2020, have the potential to enhance patient self-management. This study aimed to assess rheumatology patients’ awareness, willingness to use, suitability, and actual adoption of DiGAs. Between February 17 and April 8, 2025, adult patients attending seven German rheumatology outpatient clinics completed an electronic survey. A total of 246 patients participated (mean age 50.4 years; 71.1% female), with most treated at university hospitals (59.8%). The predominant diagnoses were rheumatoid arthritis (41.1%), psoriatic arthritis (18.3%), and axial spondyloarthritis (10.2%). While only 19.5% reported prior use of medical apps, 39.8% were aware of DiGAs, and 12.6% had used one. Notably, 84.6% reported at least one comorbidity matching an approved DiGA indication, most commonly back pain (54.8%), chronic pain (52.0%), and sleep disorders (35.8%). A majority expressed willingness to regularly use a DiGA (72.4%) and were open to recommendations from their rheumatologists or health insurers (72.8%). Additionally, 76.0% showed interest in a rheumatology-specific DiGA. Despite high interest and relevant comorbidities among patients, current DiGA use was limited. These findings underscore the need for targeted implementation strategies to increase uptake and realize the full potential of digital health applications in rheumatology care. The results also emphasize the need for rheumatologists to actively educate and guide their patients regarding the availability and potential benefits of DiGAs.

**Supplementary Information:**

The online version contains supplementary material available at 10.1007/s00296-025-05922-3.

## Introduction

Rheumatic diseases are frequently accompanied by a range of comorbidities, including cardiovascular disease, depression, anxiety, and metabolic disorders, which significantly contribute to impaired quality of life, healthcare costs [1–3], and negative effects on treatment response [4]. Despite the high prevalence of these comorbidities their management remains suboptimal in routine care [5, 6]. Barriers such as limited access to multidisciplinary care, insufficient integration of mental and physical health services, and geographic or socioeconomic constraints often hinder timely and comprehensive treatment of these comorbidities [1, 7, 8].

In order to tackle this rising problem and improve patient self-management the European Alliance of Associations for Rheumatology (EULAR) actively recommends incorporation of digital health solutions in rheumatology care [9, 10]. In recent years, there has been growing acceptance and usage of digital health applications by rheumatic patients and rheumatologists [11, 12]. Prescribable fully automated digital therapeutics offer a scalable, and potentially cost-effective solution to address these care gaps [13].

Germany was the first country to pass a law enabling prescription of digital therapeutics in 2020 [14]. DiGAs (Digitale Gesundheitsanwendung) are certified digital therapeutics that can be prescribed by physicians or psychotherapists in Germany and are reimbursed by statutory health insurance. DiGAs are native or web-based apps designed to support the detection, monitoring, treatment, or alleviation of diseases. To be listed in the official DiGA directory by the German Federal Institute for Drugs and Medical Devices (BfArM), these applications must demonstrate positive healthcare effects in trials such as medical benefits or improved patient-relevant processes and need to be medical products.

The number of available DiGAs is continuously increasing. As of April 2025, 69 DiGAs have been approved [15]. At present, no DiGA is tailored to inflammatory rheumatic diseases, however key comorbidities such as chronic pain, back pain, depression, smoking, and overweight are DiGA indications [14]. Randomized controlled trials are most often carried out for BfArM approval of DiGAs. Most approved DiGAs focus on mental health, particularly depression, with primary outcomes typically assessing traditional medical benefits such as symptom improvement [16]. Promising DiGAs have also been approved for use in otorhinolaryngology [17] and gynecology [18]; however, clinical adoption has remained limited [17–20]. Key barriers include a lack of real-world evidence, low patient adherence, the time required to onboard patients, and concerns about pricing raised by physicians [21]. While an increasing body of real-world evidence supports the effectiveness of DiGAs in rheumatology [22–24], evidence in other fields is lacking. Furthermore, the actual frequency of DiGA-indicated conditions, as well as their adoption and acceptance among patients with rheumatic diseases, remains insufficiently understood. This study aimed to address this gap by evaluating patient awareness, willingness, suitability, and adoption of DiGAs in rheumatology.

## Methods

### Study design

Between February 17 2025 and April 8 2025, consecutive patients seen at seven German rheumatology outpatient clinics were asked to complete a web-based cross-sectional REDCap (Research Electronic Data Capture; Vanderbilt University) survey. The Philipps-University Marburg Research Ethics Committee confirmed that no ethical approval was required (25–45 ANZ) for this anonymous survey study.

The survey (English translation, see supplementary file 1) was drafted considering recommendations by Zimba and Gasparyan [25], approved DiGAs as of February 15 2025, and related previous ehealth surveys in rheumatology [11, 12, 22, 23]. The survey was pretested by the coauthors. As no additional suggestions were made and the questionnaire length was considered appropriate, no further modifications were implemented. The survey collected information on participants’ age, gender, primary rheumatic diagnosis, and the treating center. It also included questions on current and past use of medical apps and DiGAs, awareness of DiGAs, and the presence of comorbidities that align with approved DiGA indications, including back pain, chronic pain, sleep disorders, stress, overweight, smoking, depression, diabetes, and alcohol abuse. Additionally, patients were asked whether they would be willing to use a DiGA for at least 10 minutes once per week, whether they would welcome personalized DiGA recommendations from their rheumatologist or health insurer, and whether they were interested in using a DiGA specifically designed for inflammatory rheumatic diseases. Results were reported according to recommendations by Zimba and Gasparyan [25]. Characteristics were summarized using means, standard deviations, counts, and percentages as appropriate. We used Excel (Microsoft Corp), R (version 3.5.3; R Foundation for Statistical Computing), GraphPad Prism (GraphPad Software Inc., San Diego, USA) and SankeyMATIC (https://sankeymatic.com) for the analyses.

## Results

### Participant characteristics

A total of 246 patients (Table 1) completed the survey. The mean age was 50.4 years (range: 20–84) and the majority were female (71.1%). Most patients were receiving rheumatologic care at a university hospital (59.8%). The primary indications for rheumatologic care included rheumatoid arthritis (41.1%), followed by psoriatic arthritis (18.3%) and axial spondyloarthritis (10.2%).

**Table 1:** Participant characteristics

Characteristic	Group	Total *n* (%)
Gender	Female	175 (71.1)
	Male	70 (28.5)
	Other	1 (0.4)
Age, years	18–39	70 (28.5)
	40–59	96 (39.0)
	≥ 60	79 (32.1)
	Mean (SD)	50.4 (15.3)
	University Hospital	147 (59.8)
	General Hospital	27 (11.0)
Rheumatology setting	Practice	67 (27.2)
Disease	Rheumatoid Arthritis	101 (41.1)
	Psoriatic Arthritis	45 (18.3)
	Axial Spondyloarthritis	25 (10.2)
	Other	66 (26.8)

## DiGA awareness, suitability, adoption and interest

**Fig. 1:**
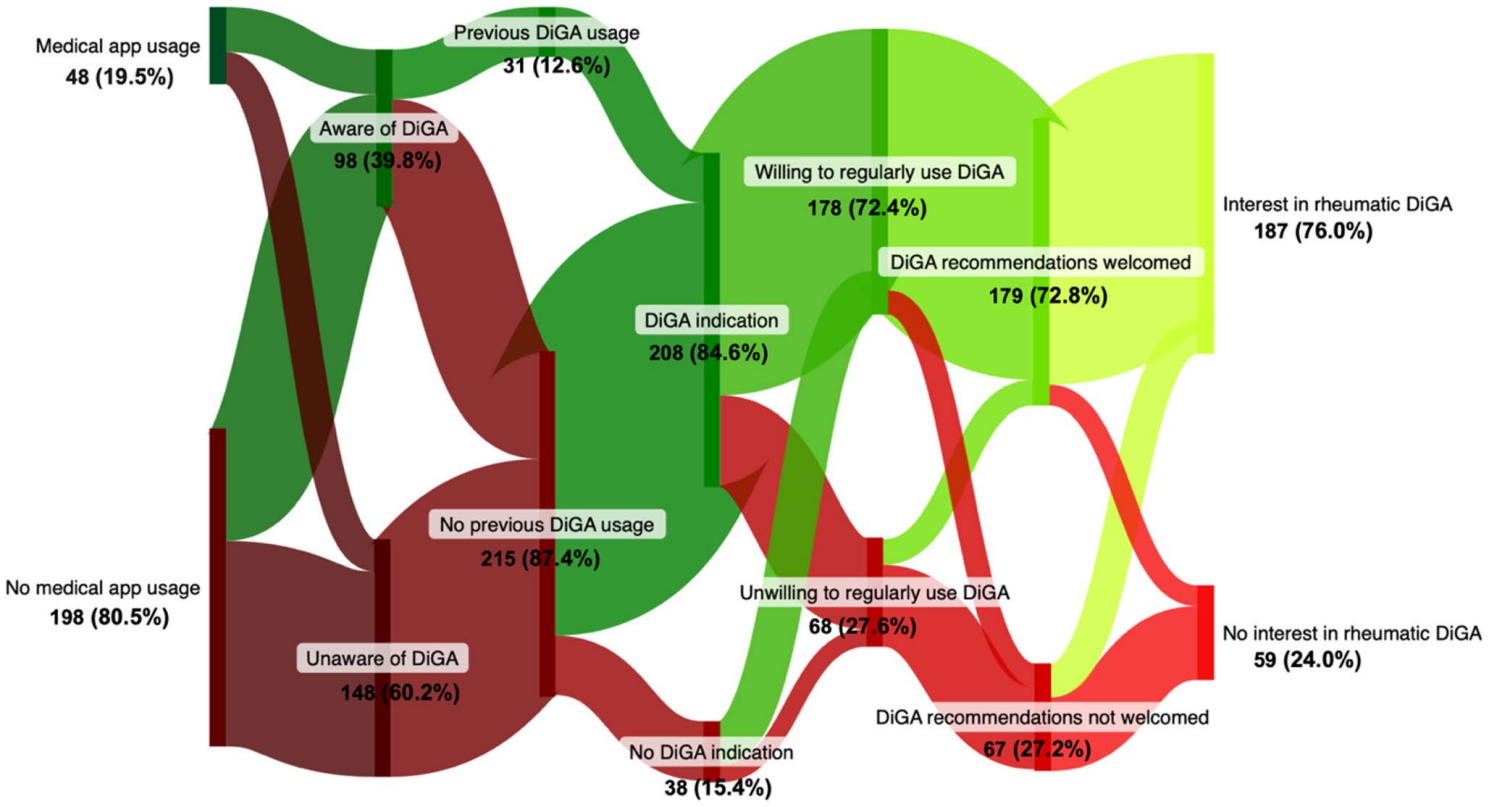
Sankey diagram illustrating patients’ medical app usage, DiGA awareness, DiGA usage, DiGA indications, willingness to use DiGAs, interest in receiving DiGA recommendations, and interest in a rheumatology-specific DiGA

19.5% of participants reported prior use of medical apps, 39.8% were aware of DiGAs, and 12.6% had previously used one (Fig. 1). A substantial proportion (72.4%) expressed a willingness to regularly use a DiGA, and 72.8% indicated openness to recommendations from their rheumatologists or health insurers. Furthermore, 76.0% of respondents expressed interest in a DiGA for inflammatory rheumatic diseases.

**Fig. 2:**
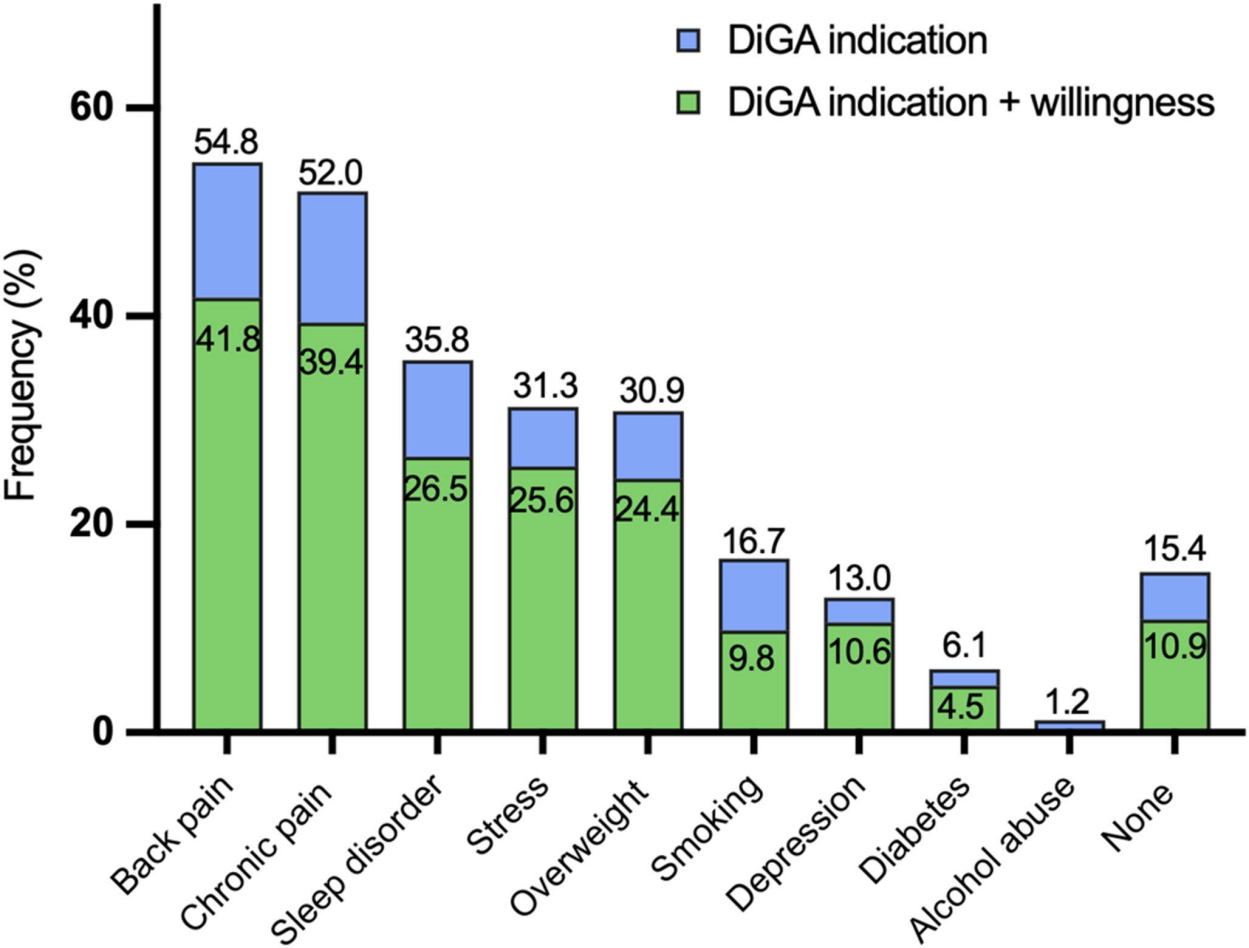
Patient-reported comorbidities that represent indications for approved prescribable digital health applications (DiGAs). Participants were allowed to select multiple comorbidities. Light blue depicts the proportion of patients with the reported respective indication. Green depicts the proportion of patients with the reported respective indication and reported willingness to regularly use a DiGA

Notably, 84.6% reported at least one comorbidity matching an approved DiGA indication, most commonly back pain (54.8%), chronic pain (52.0%), and sleep disorders (35.8%), see Fig. 2.

## Discussion

The aim of this study was to assess patient awareness, willingness, suitability, and actual use of DiGAs in rheumatology. Despite the availability of DiGAs for managing various comorbidities, their adoption among patients remained low, with only 12.6% reporting prior use in this study. This finding underscores a significant gap between the potential of DiGAs and their real-world clinical integration. Notably, a substantial proportion of patients expressed interest in using DiGAs, in line with previous studies and a clear preference for receiving recommendations from their rheumatologists or health insurers [11, 12].

However, this patient demand is unlikely to be fully met under current conditions. According to a 2021 survey, only a minority (7%) of German rheumatologists had prescribed a DiGA, and approximately 30% were unaware that they were authorized to do so [20]. While more recent data from 2024 suggest a growing uptake of DiGAs among internal medicine physicians in Germany, these findings also emphasize the ongoing need for targeted education and awareness initiatives among healthcare providers, including rheumatologists [19]. Encouragingly, the majority of surveyed physicians believed that DiGAs have the potential to enhance patient health. A second major barrier to DiGA adoption previously identified is the limited time available to rheumatologists for patient education [26]. Sustainable DiGA integration will likely require structural delegation strategies, such as embedding DiGA counselling into routine workflows supported by rheumatology nurses, physician assistants, or digital onboarding tools. Stakeholder interviews have identified several additional macro-level changes that could support DiGA implementation beyond rheumatology, including broader dissemination of information, enhanced financial incentives, streamlined digital prescription and activation processes, and increased adoption of blended care models and pay-for-performance approaches [26, 27]. Furthermore, the approval of a DiGA specifically designed for inflammatory rheumatic diseases could significantly enhance overall DiGA adoption within rheumatology [14].

Interestingly, the most frequently reported comorbidity, potentially addressable with DiGAs was back pain. Notably, real-world data from the DiGAReal registry indicate that back pain–specific DiGAs account for the highest proportion of patients reporting symptom improvement, including significant reductions in pain levels [23]. Consistent with these findings, earlier real-world analyses have also identified back pain DiGAs as the most effective group among DiGAs presecribed to rheumatic patients [22].

The cross-sectional design and reliance on self-reported data represent the primary limitations of this study. Additionally, since survey completion required digital literacy and access to electronic devices, the study sample may overrepresent digitally engaged patients, potentially leading to an overestimation of DiGA interest. As a result, the findings may not be fully generalizable, and actual DiGA usage may differ from self-reported behavior. Nevertheless, a notable strength of the study lies in its inclusion of multiple sites across Germany, encompassing both university-affiliated and non-university outpatient clinics.

## Conclusion

This study highlights a substantial gap between the high prevalence of DiGA-relevant comorbidities and the limited use of DiGAs among patients with inflammatory rheumatic diseases. Although patient interest and willingness were strong, actual adoption remained low.

Targeted efforts are needed at multiple levels, including provider education, delegation strategies and development of rheumatology-specific DiGAs, to convert patient interest into real-world use and improved patient outcomes.

## Electronic supplementary material

Below is the link to the electronic supplementary material.

Supplementary Material 1

## Data Availability

The raw data supporting the conclusions of this article will be made available by the authors upon reasonable request.
