# Supplementary material for: Digital empowerment on hold: DiGA adoption gaps−a German national cross-sectional patient survey study
Source: Rheumatol Int. 2025 Jul 4;45(7):165. doi: 10.1007/s00296-025-05922-3 (PMC12227445; doi:10.1007/s00296-025-05922-3)
Supplement: Supplementary file 1 — Supplementary Material 1 [file 296_2025_5922_MOESM1_ESM.docx]

# Survey on Digital Health Applications in Rheumatology

Thank you very much for supporting us by completing this short survey.

Gender:

☐ Female
☐ Male
☐ Other

Age:

__________________________________
(in years, e.g., 35)

Where are you primarily receiving rheumatologic care?

☐ University hospital
☐ Other hospital
☐ Private practice

Rheumatic disease:

☐ Rheumatoid arthritis
☐ Psoriatic arthritis
☐ Axial spondyloarthritis
☐ Gout
☐ Polymyalgia rheumatica
☐ Polyosteoarthritis
☐ Other disease

Please name the disease:

__________________________________

Please select what applies to you:

☐ I suffer from back pain
☐ I am very stressed
☐ I suffer from chronic pain
☐ I smoke
☐ I am overweight
☐ I have trouble falling/staying asleep
☐ I am depressed
☐ I have diabetes
☐ I drink too much alcohol
☐ None of the above

Do you already use medical apps?

☐ Yes
☐ No

Have you heard of Digital Health Applications “prescription apps” before?

☐ Yes
☐ No

Have you already used Digital Health Applications “prescription apps”?

☐ Yes
☐ No

Can you imagine using a DiGA at least once per week for at least 10 minutes?

☐ Yes
☐ No

Would you like to be informed by your doctor or health insurer about DiGAs suitable for you?

☐ Yes
☐ No

Would you be interested in a rheumatology-specific DiGA (e.g., information, tracking, exercises)?

☐ Yes
☐ No
